# A dual-database bibliometric analysis of music-based interventions and pain from 2004 to 2024

**DOI:** 10.3389/fmed.2025.1671234

**Published:** 2025-10-16

**Authors:** Xianghui Zou, Ziqi Jin, Weiqing Zeng

**Affiliations:** ^1^Guilin University of Aerospace Technology, Guilin, Guangxi, China; ^2^Graduate College, Guangxi University of Chinese Medicine, Nanning, Guangxi, China; ^3^Department of Orthopedics, Guilin Municipal Hospital of Traditional Chinese Medicine, Guilin, Guangxi, China

**Keywords:** music therapy, pain management, analgesia, databases, bibliometrics

## Abstract

**Objectives:**

To explore the research hotspots and trends in the field of music therapy and pain based on bibliometrics and to provide reference and guidance for the current status and development of research in this field.

**Methods:**

Using the Web of Science Core Collection (WOSCC) and Pubmed databases as data sources, we used VOSviewer and CiteSpace, R language combined, way to visualize and analyze the number of publications, countries, institutions, researchers, keywords, and literature in the fixed field from 2004 to 2024.

**Results:**

Publication volume increased each year, with 2013 having the highest average citation rate. Lead authors included Bradt Joke, Silverman Michael J., Warth Marco, and Kessler Jens. the Journal of Music Therapy ranked first in terms of published papers. The United States leads in research but lacks national collaboration. Research topics have evolved from anxiety, cancer, and pain management to rehabilitation, virtual reality, and quality of life.

**Conclusion:**

This study presents a roadmap for optimizing the clinical application of music therapy, addresses gaps in protocol standardization, advocates for cross-national collaboration to improve research quality, and provides important insights and guidance for future music therapy and pain research and applications.

## 1 Introduction

In recent decades, substantial progress in pain research has highlighted its dual nature as both an emotional perception and a sensory experience associated with tissue pathology. Since 2020 the International Association for the Study of Pain has defined pain as “an unpleasant sensory and emotional experience associated with, or resembling that associated with, actual or potential tissue damage” (1). This dual-character taxonomy explicitly separates the sensory-discriminative component from the emotional–motivational component. Chronic pain now exceeds the combined disability-adjusted life years (DALYs) attributable to diabetes, ischemic heart disease and depression (2). It is estimated that more than 20% of the population seeks medical attention annually, citing pain as their principal complaint (3). The economic burden of pain is significant, representing roughly 3% of GDP (Gross Domestic Product) (4). Pain management remains complex due to heterogeneous aetiologies and clinical presentations. Contemporary classification systems categorize pain by duration (acute/chronic), mechanism (nociceptive/neuropathic), anatomical location, and underlying pathology. Although NSAIDs, muscle relaxants, and anticonvulsants offer temporary relief, their prolonged use is frequently constrained by side effects and the development of tolerance. Consequently, multimodal strategies that combine optimized pharmacotherapy with physical rehabilitation and psychological interventions are increasingly advocated to improve treatment efficacy and reduce complications. Moreover, emerging non-pharmacological interventions (e.g., neuromodulation, image-guided ablation) demonstrate improved safety profiles, especially in the management of neuropathic pain. Contemporary guidelines emphasize personalized pain management through integrated strategies such as pain phenotyping and evidence-based escalation protocols (5). Among these strategies, music therapy—a clinical modality delivered by certified therapists (6) —is increasingly implemented within rehabilitation, public health, and psychiatry to augment conventional care (7). Music therapy is the clinical and evidence-based use of music interventions to accomplish individualized goals within a therapeutic relationship by a credentialed professional who has completed an approved music therapy programme (8). In recent years, investigations into music therapy for pain have proliferated, resulting in an increasing volume of published studies examining its therapeutic efficacy (9, 10). This study presents a thorough bibliometric analysis of research on music therapy for pain, delivering both a wide-ranging overview and a detailed exploration of the field’s knowledge framework. This study delineates the evolution of music therapy research in pain management through an analysis of global collaboration and contributions from countries, institutions, and authors. Furthermore, keyword co-occurrence analysis and comprehensive literature reviews are utilized to pinpoint research hotspots. An analysis of trending themes further investigates emerging frontiers and prospective future directions in this domain (11). This analysis provides significant insights into the changing dynamics of music therapy for pain, highlighting essential trends and guiding future research and clinical applications.

## 2 Materials and methods

### 2.1 Data collection and retrieval strategy

Data from the Web of Science Core Collection (WOSCC) database of the library of Guangxi University of Chinese Medicine were used for bibliometric analysis. WOSCC was chosen for its citation network compatibility with bibliometric tools with the following search strategy (12): [TS = (“music therapy”) OR TS = (“Music Therapy”) OR TS = (“Musical Therapy”) OR TS = (“Therapeutic Musical”) OR TS = (“Music-Mediate Therapy”) OR TS = (“Clinical Music Therapy”) OR TS = (“Music, Therapy”) OR TS = (“music treatment”) OR TS = (“musical intervention”) OR TS = (“Musical Intervention”)] AND [TS = (“Pain”) OR TS = (“Ache”) OR TS = (“Soreness”) OR TS = (“Sore”)]. PubMed was subsequently queried solely for clinical-trial data to verify whether the temporal trends observed in WOSCC were reproducible in a medically focused repository. PubMed was used with the search strategy: {[music therapy (Title/Abstract)) OR [musical intervention (Title/Abstract)] OR [Therapeutic Musical (Title/Abstract)]} AND [pain (Title/Abstract)]. We selected WOSCC—with its broad disciplinary coverage and diverse document types—as the primary data source for the overarching bibliometric analysis, and complemented it with PubMed, a medically specialized repository, to verify trends within clinical-trial literature. This dual-source strategy ensures that our project simultaneously captures the full breadth of research activity (via WOSCC) and the targeted depth of clinical evidence (via PubMed).

### 2.2 Inclusion and exclusion criteria

**FIGURE 1:**
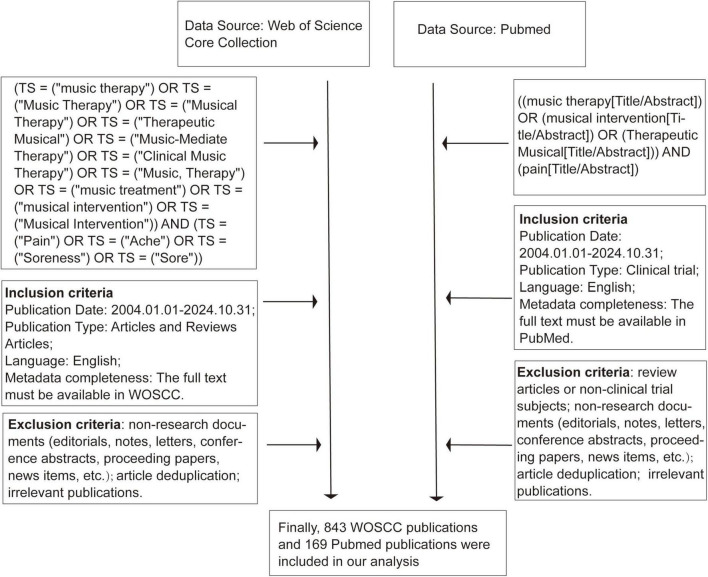
Flowchart of study selection from publications.

The two datasets were available up to 31 October 2024, and covered publications from 2004 to 2024, especially full metadata completeness English publications. Only documents categorized as articles or review articles were included in the WOSCC dataset. PubMed was chosen for clinically focused literature from MEDLINE-indexed journals and clinical trial reports. Excluded from our analysis of two datasets were editorials, notes, letters, conference abstracts, proceeding papers, book reviews, book chapters, news items, early accesses, corrections, and retracted publications. Additionally, review articles or non-clinical trial subjects were excluded in PubMed, ensuring the relevance of our findings focused on clinical-trial progress. Two researchers independently examined the title, abstract, authors, and keywords of each article to determine its relevance to music-based interventions and pain, excluding non-medical uses or irrelevant articles, and any duplicates were removed to ensure that every retained article was unique. In cases of disagreement between the two researchers, a third person made the final decision. Finally, we found 843 relevant articles or review articles from the WOSCC database, and 169 clinical trial reports were found from the PubMed database (Figure 1).

### 2.3 Data analysis

The data were processed with the Bibliometrix R package (v4.4.1), CiteSpace (6.3.R1 Pro), and VOSviewer (1.6.20). The Bibliometrix R package (R4.4.1) is a commonly employed tool for quantitative bibliometric analysis. The literature data obtained from WOSCC were imported into Bibliometrix R (R4.4.1) to perform various analyses, including H-index evaluations for journals and authors, assessments of institutional publication output and H-index, country-specific distribution and collaboration networks, institutional affiliations, keyword trends, thematic patterns, and literature emergence for visualization and analysis.

CiteSpace was widely utilized in medical informatics to identify emerging trends in particular research domains. CiteSpace visualizations incorporate data rings and lines, with ring size denoting the frequency of occurrence or citation of a specific paper, and color variations signifying the publication year. The width of the rings represents citation counts over time, while the connecting lines depict citation relationships, with line width indicating citation strength (Figure 1). This study utilized CiteSpace (6.3.R1 Pro) to visualize and analyze institutional collaborations and literature co-citation networks chronologically.

VOSviewer is a freely accessible software application used mainly for bibliometric network analysis. It generates and illustrates diverse data maps, encompassing author and journal maps based on co-citation data, in addition to keyword maps derived from co-occurrence data (13). The maps produced in VOSviewer comprise data rings and connecting lines, with the size of the rings corresponding to the frequency of occurrence or citation of an author’s published works. The ring color signifies the research team affiliation of an author, whereas the connecting lines denote relationships such as co-authorship. This study used VOSviewer (1.6.20) to visualize and analyze author collaboration networks and keyword clustering patterns.

## 3 Results

### 3.1 Annual publication and publication trend analysis from the WOSCC database

**FIGURE 2:**
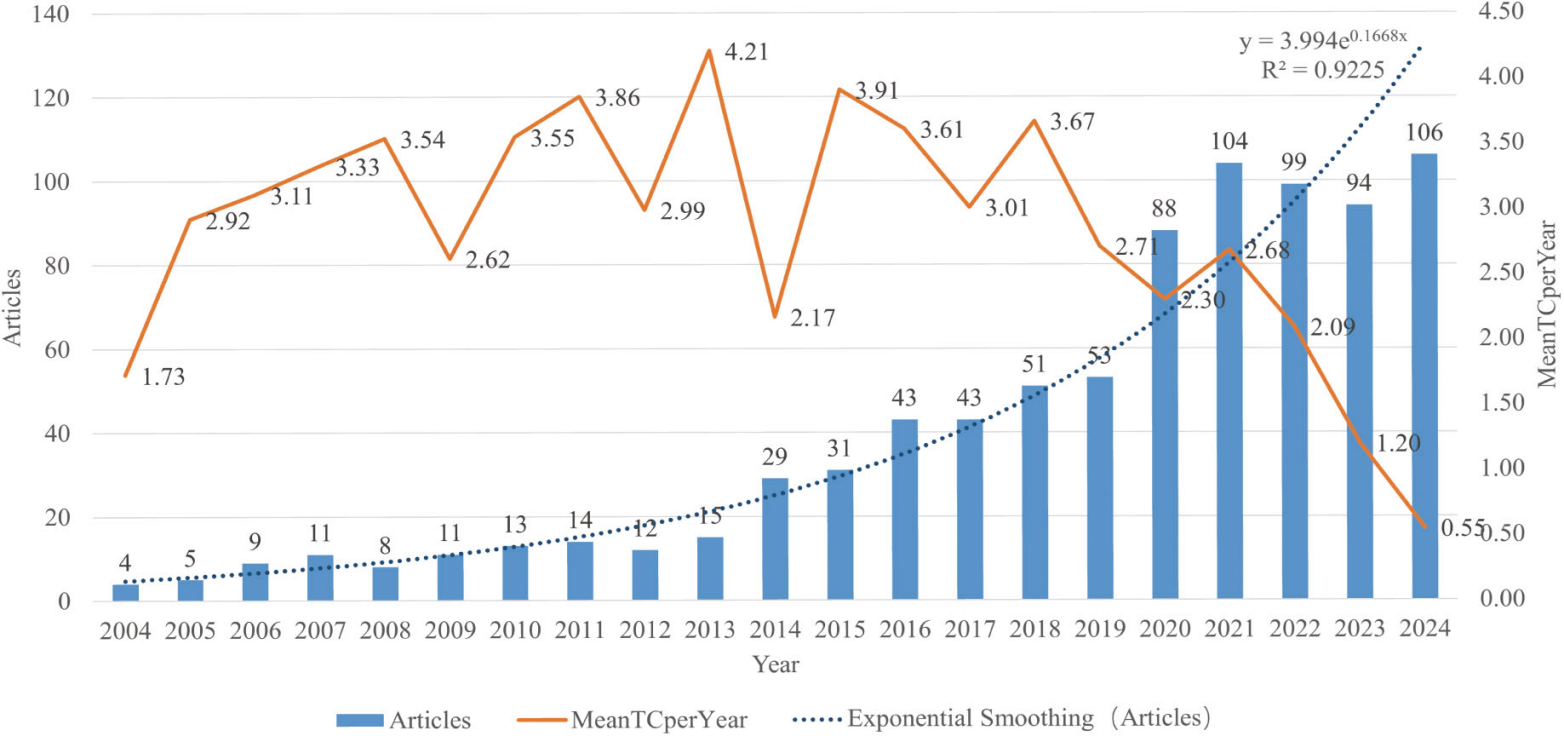
Annual publications addressing music therapy and pain and their yearly number of citations in the database from 2004 to 2024.

A total of 843 articles on music therapy and pain were retrieved from the Web of Science Core Collection (2004–2024) and subjected to bibliometric analysis (Figure 2). The number of annual publications in this field increased by 26.5 times from 2004 to 2024, rising from 4 to 106 articles. Post-2020 growth accelerated markedly, with annual outputs exceeding 80 articles—an 81% increase compared to the 2015–2019 baseline period (mean = 44.2 articles/year). This trajectory underscores sustained prioritization of the research focus. Citation analysis revealed temporal variations in academic impact. The highest average citation rate (4.2 citations/year) was observed for 15 articles published in 2013, indicating their enduring influence. Interrupted-time-series confirms that this inflection coincided with three sequential policy levers: the CMS telehealth reimbursement waiver (March 2020), the WHO scoping document endorsing arts interventions for COVID-19 distress (July 2021), and the AMTA release of new CPT codes for virtual music therapy (December 2022). These events, rather than purely academic interest, appear to have been the major drivers of the sustained surge in publication activity.

### 3.2 Analysis of authors and their H-index in the WOSCC database

**FIGURE 3:**
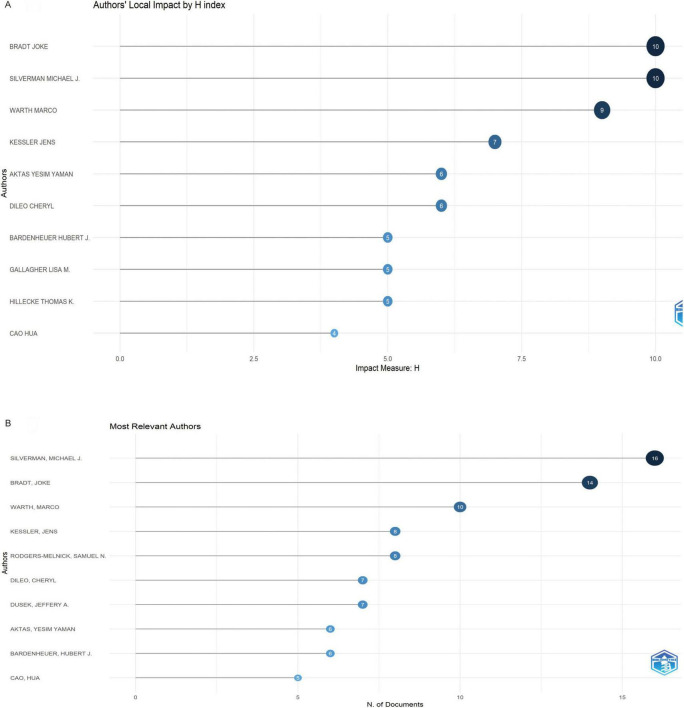
**(A)** Top 10 authors by H-index in music therapy and pain research. **(B)** Number of articles by authors.

A total of 3,634 authors contributed to music therapy and pain research, with 15 authors publishing at least five articles. The top three authors ranked by H-index were led by Bradt Joke (H = 10), Silverman Michael J. (H = 10), and Warth Marco (H = 9) (Figure 3A). Notably, four authors—Bradt Joke, Silverman Michael J., Warth Marco, and Kessler Jens—demonstrated concurrent prominence in both publication volume and academic impact (Figure 3B and Table 1).

**TABLE 1:** Top 10 authors in terms of number of articles published.

Rank	Authors	Articles	H-index	Country
1	Silverman, Michael J.	16	10	USA
2	Bradt, Joke	14	10	USA
3	Warth, Marco	10	9	Germany
4	Kessler, Jens	8	7	Germany
5	Rodgers-Melnick, Samuel N.	8	7	USA
6	Dileo, Cheryl	7	6	USA
7	Dusek, Jeffery A.	7	6	USA
8	Aktas, Yesim Yaman	6	6	Turkey
9	Bardenheuer, Hubert J.	6	5	Germany
10	Cao, Hua	5	4	Canada

**FIGURE 4:**
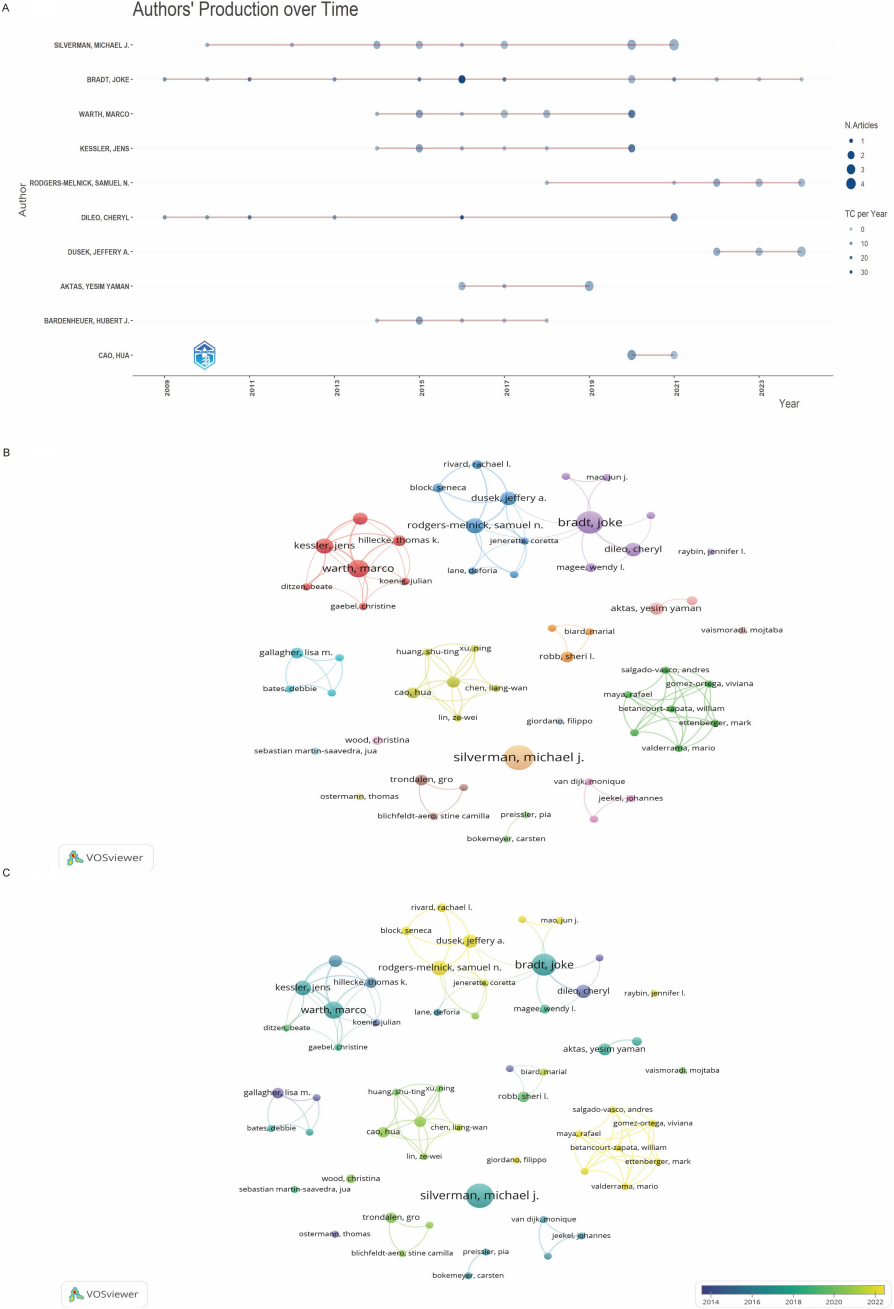
**(A)** Publication volume and citation frequency of high-yield authors across years. **(B)** Author co-authorship network. **(C)** Timeline of author collaborations.

Analysis of annual publication and citation patterns among high-productivity authors (Figure 4A) revealed distinct chronological phases. No contributions from these authors were recorded between 2004 and 2008. Bradt Joke and Dileo C emerged as pioneers in 2009, establishing sustained research outputs, while Warth Marco and Kessler Jens (co-affiliated researchers) began collaborative work in 2014. Dusek JA entered the field later in 2022 but achieved rapid productivity. Bradt Joke’s 2016 article accumulated the highest citations (*n* = 127), highlighting its foundational influence, whereas Cao H’s contributions were limited to 2020–2021. Robb SL maintained consistent scholarly engagement, contributing five indexed publications that have collectively accrued 252 citations, underscoring her sustained impact on music therapy research throughout this period.

Collaboration networks mapped by VOSviewer (Figure 4B) indicated limited cross-team interactions among leading researchers. Most partnerships occurred within isolated clusters, with minimal exceptions such as sporadic collaborations between Bradt J’s and Rodgers-Melnick SN’s groups. Recent years (2020–2024) witnessed intensified collaborations, visualized by yellow nodes (Figure 4C).

### 3.3 Journal and periodical H-index analysis

**FIGURE 5:**
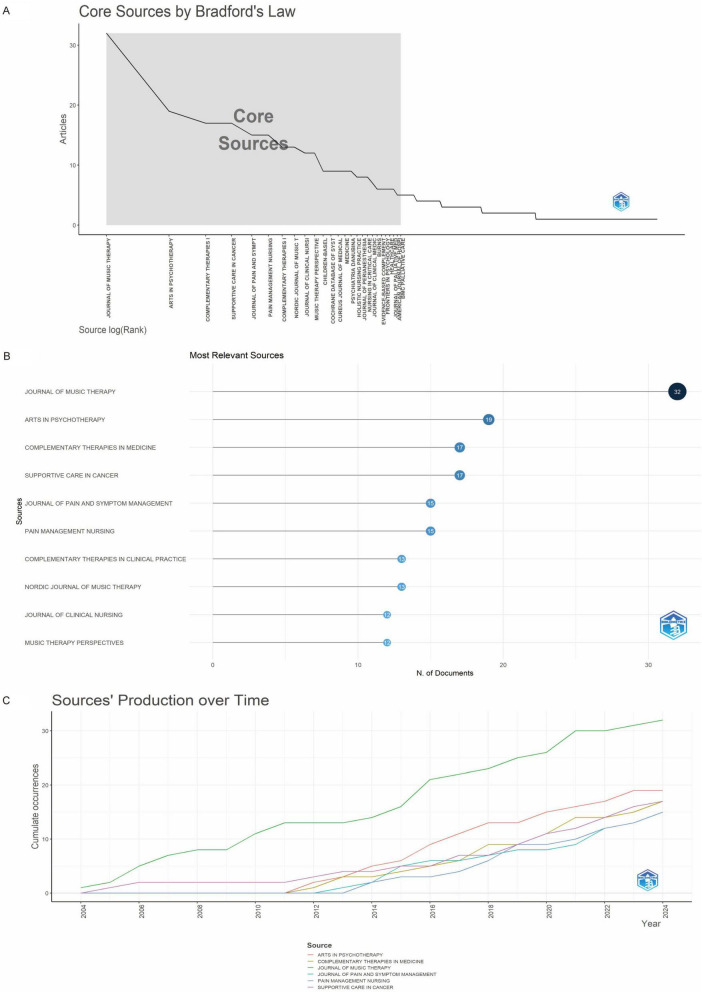
**(A)** Number of journals with publications in the field. **(B)** Top 10 journals by publication count. **(C)** Annual publication trends of the top 6 journals.

**FIGURE 6:**
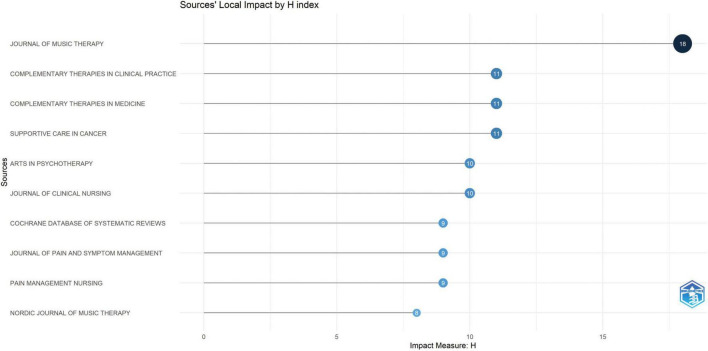
Top 10 journals by H-index among 444 journals.

A total of 444 journals published articles on music therapy and pain between 2004 and 2024 (Figure 5A; Supplementary Table 1). The top 5.9% of journals (*n* = 26) accounted for one-third of total publications. Leading journals by publication volume included Journal of Music Therapy (*n* = 32), Arts in Psychotherapy (*n* = 19), Complementary Therapies in Medicine (*n* = 17), and Supportive Care in Cancer (*n* = 17) (Figure 5B). The Journal of Music Therapy pioneered research in this field with articles from 2004, followed by Supportive Care in Cancer in 2005. Other prominent journals entered the domain post-2012. Journal of Music Therapy maintained consistent publication dominance throughout the study period (Figure 5C). The H-index ranking further solidified Journal of Music Therapy’s leadership, as it topped both publication volume and citation impact metrics (Figure 6).

### 3.4 Analysis of H-index of journals and magazines in the WOSCC database

**FIGURE 7:**
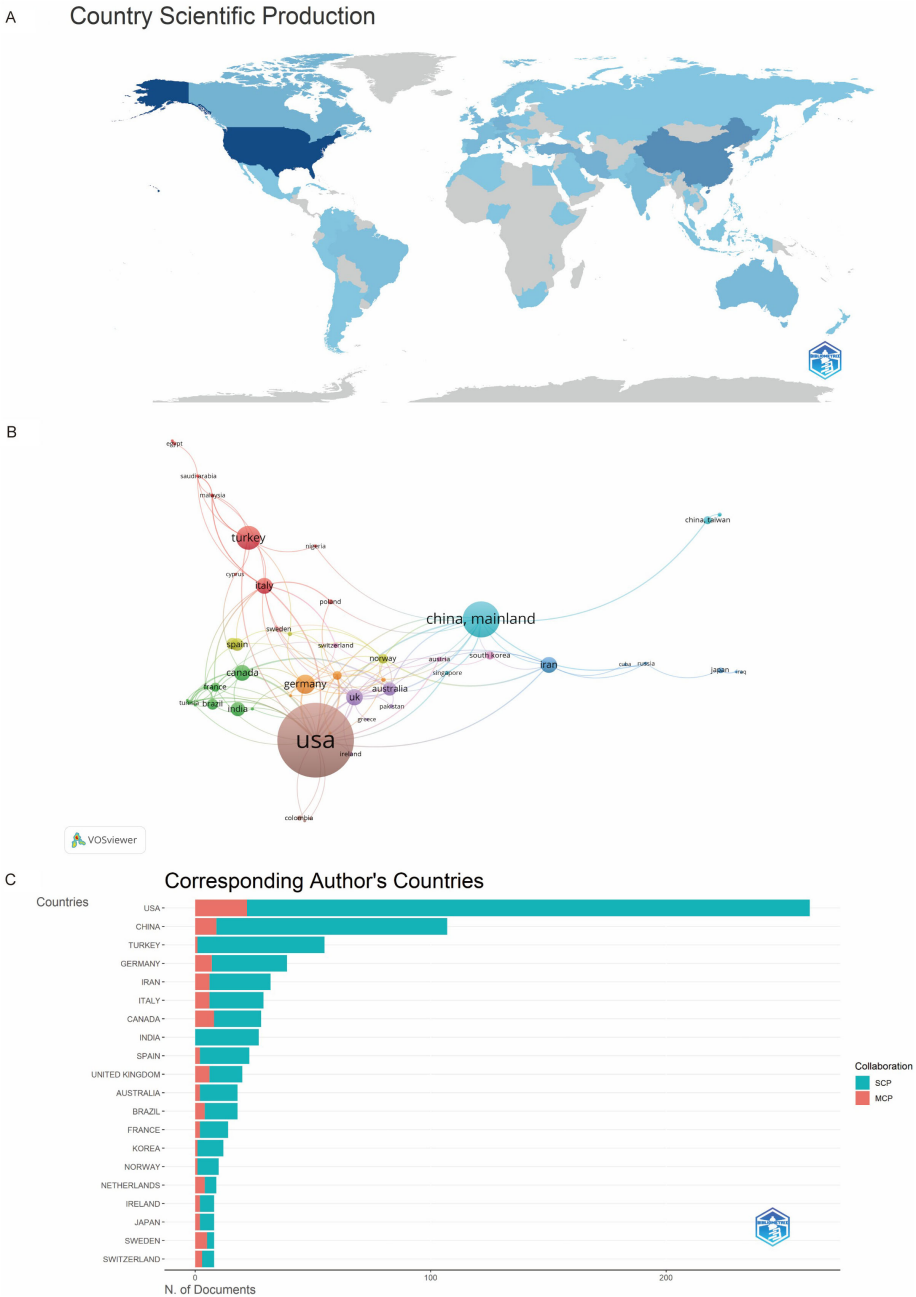
**(A)** National publication counts. **(B)** Country collaboration network. **(C)** National article counts (MCP, multi-country publications; SCP, single-country publications).

**FIGURE 8:**
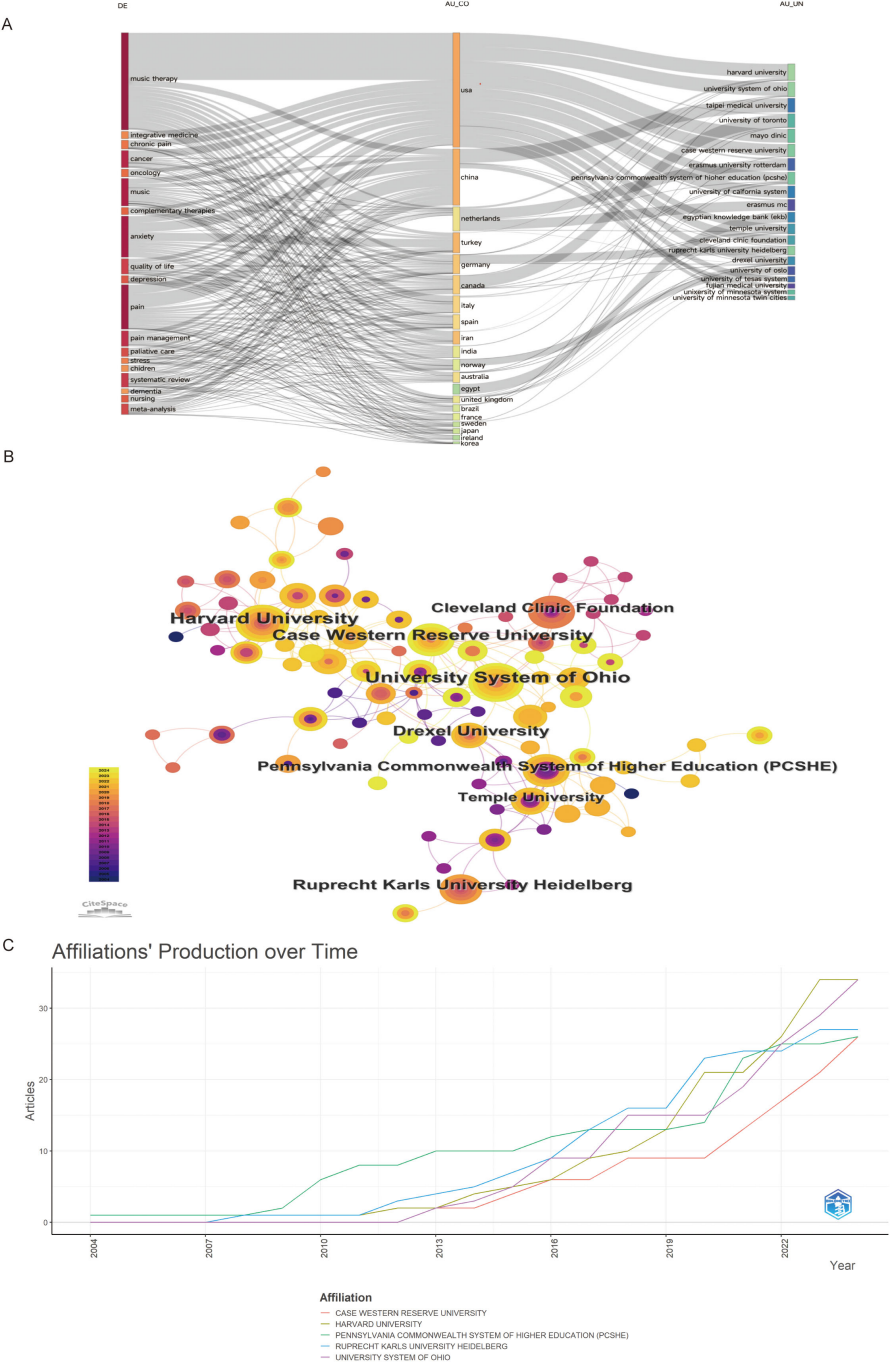
**(A)** Three-field diagram: country (middle), keywords (left), and institutions (right). **(B)** Institutional collaboration network. **(C)** Annual publication counts by institution.

Global contributions to music therapy and pain research were analyzed by country and institution. The United States (US), China, and Türkiye led in publication volume, followed by Germany, Iran, Italy, Canada, Portugal, India, Spain, and the United Kingdom (UK) (Figure 7A and Table 2). The US, China, and Canada ranked highest in citation frequency (Figure 7B). International collaboration patterns (Figure 7C) revealed concentrated partnerships among the US, China, Canada, and Germany, with limited diversity in cross-national alliances. Nine US-based and one German institution comprised the top 10 by publication volume (Figure 8A and Table 3). Institutional collaborations predominantly occurred within the US and Germany (Figure 8B). Longitudinal analysis (Figure 8C) indicated sustained research activity on music therapy and pain since 2012 among the top five institutions.

**TABLE 2:** Top 10 countries with the highest number of publications and citations.

Country	Publications	Country	Citations
USA	261	USA	6,147
China	107	China	1,338
Turkey	55	Canada	1,009
Germany	39	Germany	955
Iran	32	Turkey	815
Italy	29	Italy	420
Canada	28	Sweden	355
India	27	Korea	317
Spain	23	Australia	290
United Kingdom	20	France	269

**TABLE 3:** Top 10 organizations in terms of number of articles published.

Rank	Affiliation	Articles	Country
1	Harvard University	34	USA
2	University System of Ohio	34	USA
3	Ruprecht Karls University Heidelberg	27	Germany
4	Case Western Reserve University	26	USA
5	Pennsylvania Commonwealth System of Higher Education	26	USA
6	Mayo Clinic	25	USA
7	University of Minnesota System	25	USA
8	University of Toronto	25	Canada
9	University of Minnesota Twin Cities	23	USA
10	Cleveland Clinic Foundation	22	USA

### 3.5 Analysis of important documents, keywords and thematic trends in the WOSCC database

**FIGURE 9:**
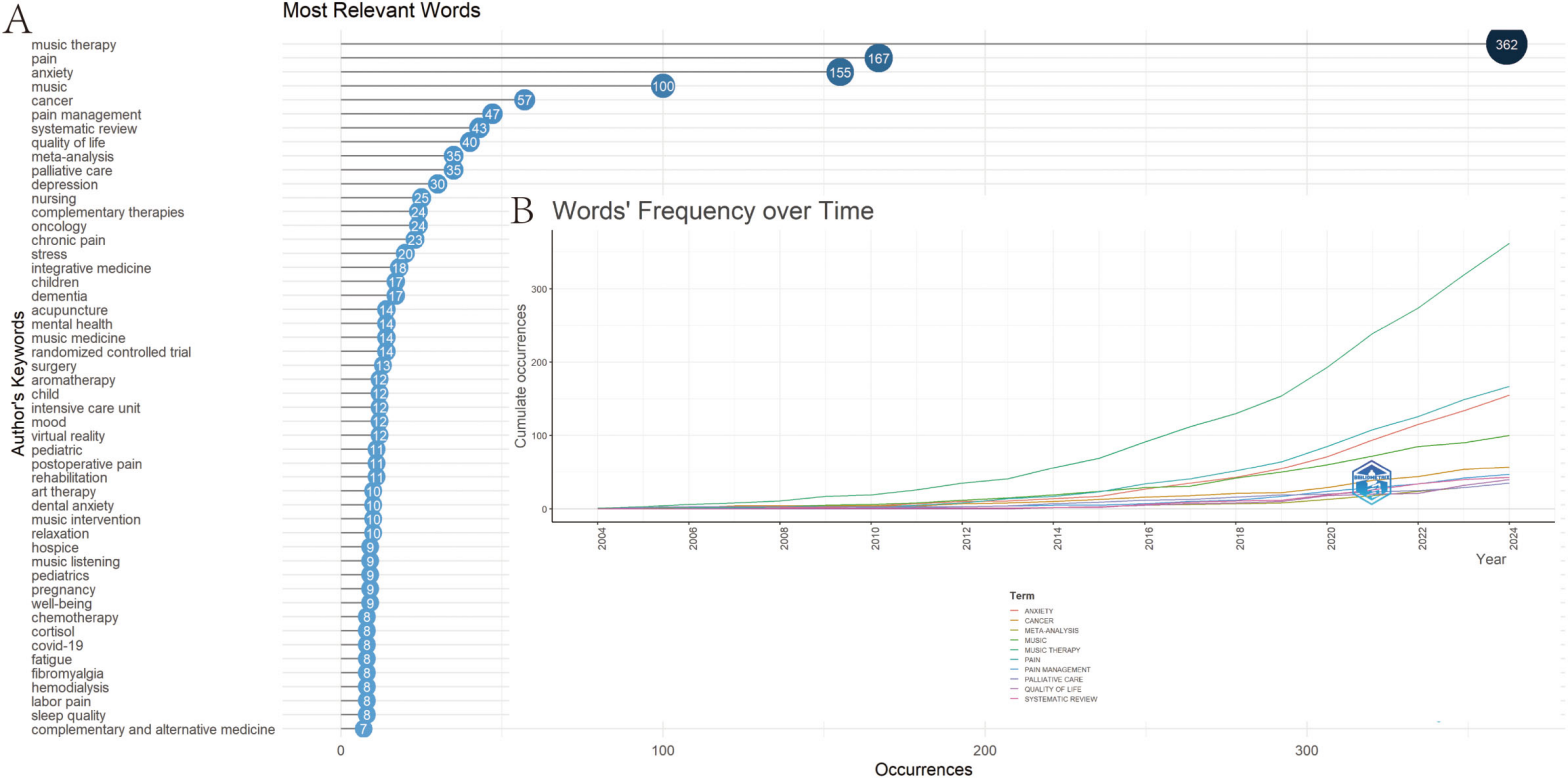
**(A)** Bibliometric keyword analysis identified dominant research themes in music therapy and pain—anxiety, cancer, pain management, systematic review, quality of life, meta-analysis, and palliative care—in both author keywords and temporal word-frequency trends. **(B)** The corresponding Words’ Frequency over Time trends for these ten keywords from 2004 to 2024.

**FIGURE 10:**
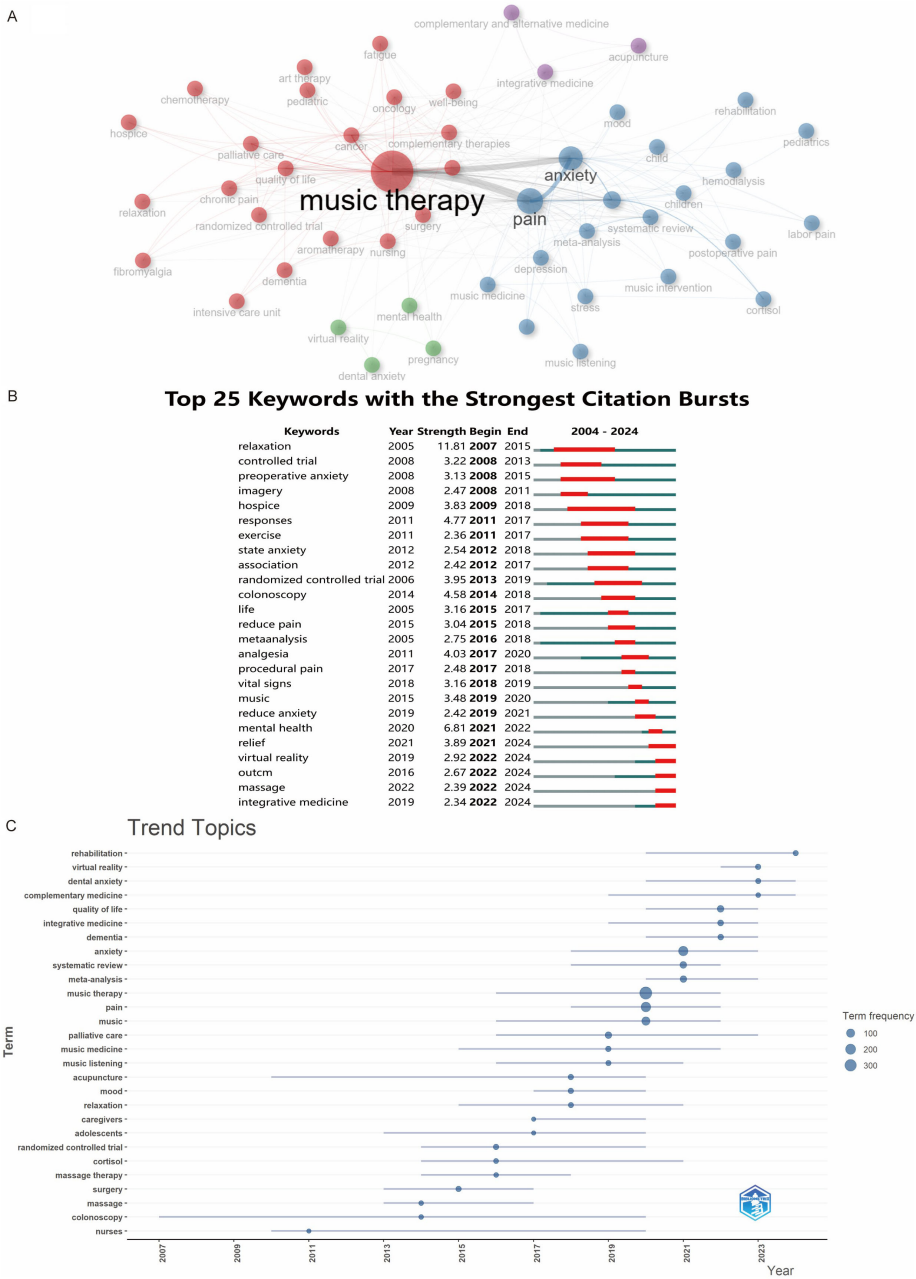
**(A)** Keyword cluster analysis. **(B)** Keyword prominence map. **(C)** Temporal trends of thematic keywords.

**FIGURE 11:**
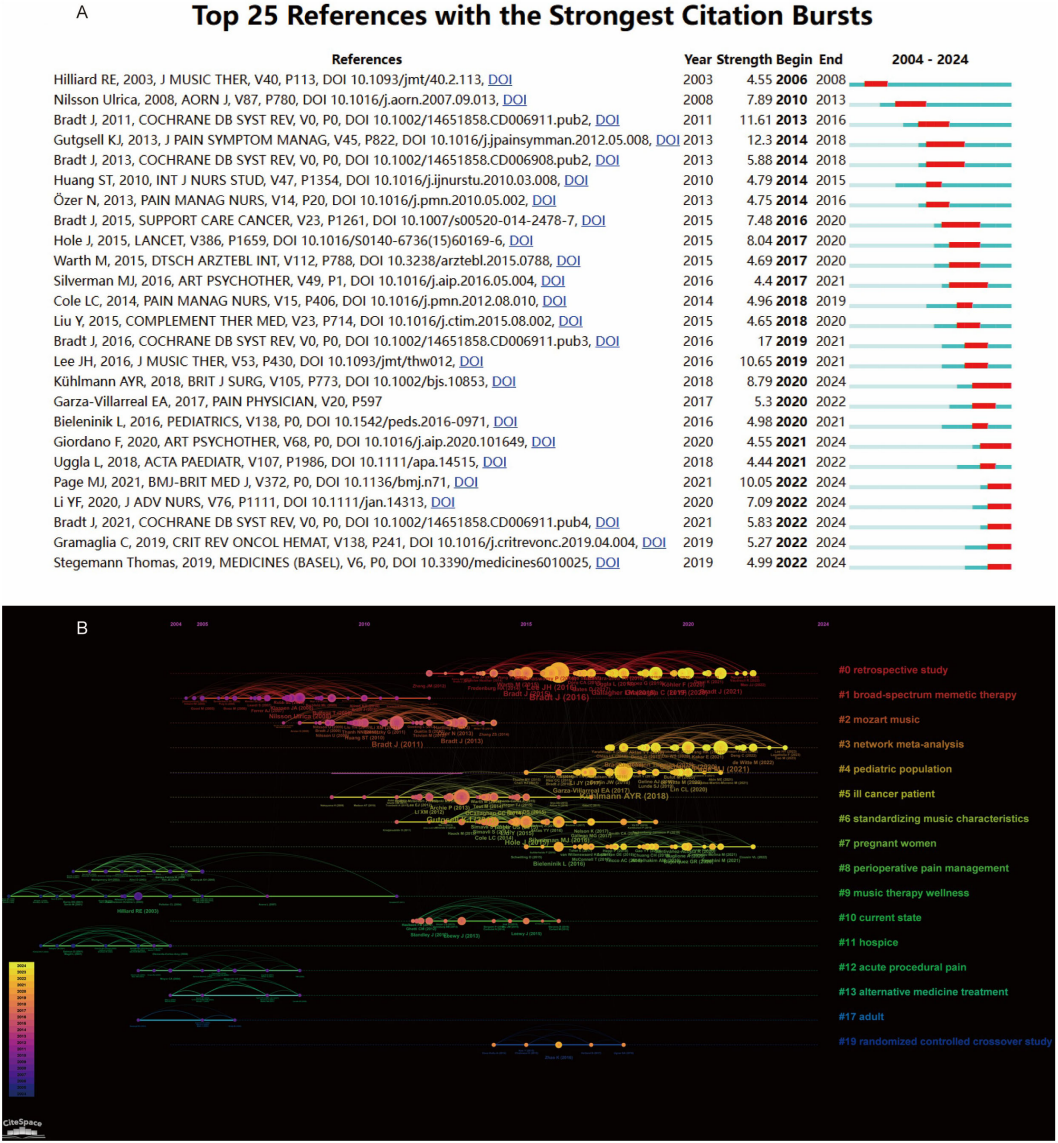
**(A)** Citation burst analysis of documents via CiteSpace. **(B)** Chronological co-citation network of literature.

Bibliometric keyword analysis identified dominant research themes in music therapy and pain—anxiety, cancer, pain management, systematic review, quality of life, meta-analysis, and palliative care—in both author keywords and temporal word-frequency trends (Figures 9A, B). Cluster analysis revealed four thematic domains: (1) Clinical Applications—music therapy for palliative care (cancer) and pain management (labor/postoperative pain, delivery/postoperative pain, children, fibromyalgia, anxiety and dementia); (2) Mental Health—anxiety reduction (preoperative/state anxiety) and cognitive support; (3) Methodological Approaches—systematic reviews, RCTs (Randomized Controlled Trials), and meta-analyses; (4) Integrative Therapies—complementary modalities like massage and Virtual Reality (VR) (Figure 10A). Temporal trends showed evolving priorities: early research focused on relaxation and hospice care, while recent studies emphasized VR, dental anxiety, and integrative rehabilitation (Figures 10B, C). Notably, combined therapies (e.g., massage + VR) remained underexplored despite growing interest post-2020. Through co-cited literature analysis, multiple representative studies have been identified. Researchers may cite pertinent literature via DOI (Digital Object Identifier) to attain a more thorough comprehension of the discipline (Figure 11A). Figure 11B shows extracted keywords from referenced literature, delineating distinct research clusters organized by magnitude and temporal sequence. Cluster #0, titled “Retrospective Studies,” is the most recent and largest cluster. Other notable and developing clusters comprise #2 Mozart music, #3 meta-analysis, #4 pediatric populations, #5 cancer patients, #6 standardized music profiles, #7 pregnant women, and #8 perioperative pain management.

### 3.6 Analysis of clinical experiments in the PubMed database

**FIGURE 12:**
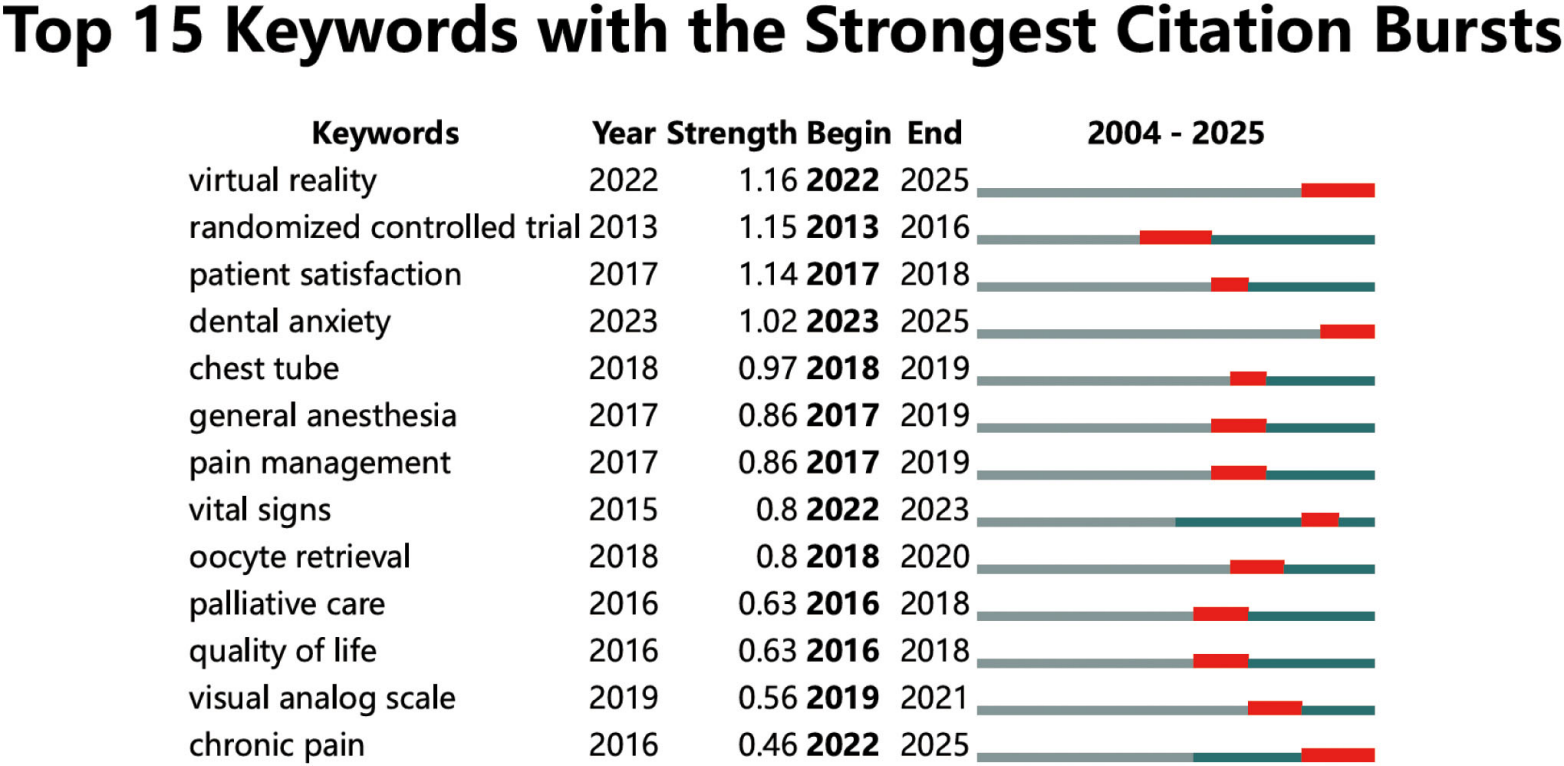
Keyword prominence analysis of clinical trials in the PubMed database.

A PubMed search covering 2004–2024 identified 169 English-language clinical trial reports that met our inclusion criteria. PubMed independently verified the trends presented by WOSCC and revealed the increase in adoption rates, including ‘virtual reality” and “dental anxiety,” among others. The specific treatment targets in this field mainly involve pediatrics and intensive care. The main research method is “visual scales.” Mechanism research is still mainly at the stage of tracking unique biomarkers, namely, “vital signs” (Figure 12). Among the 169 PubMed trials, 68% target procedural anxiety in pediatrics or special-needs adults, indicating that the bibliometric spike reflects an evidence-based expansion into acute-care settings.

## 4 Discussion

### 4.1 Contemporary overview of research in music therapy and pain management over the past twenty years

This study conducted a 20-year bibliometric analysis of the application of music therapy in pain management. The number of published papers each year generally showed an upward trend, and the output in the past 4 years remained above 80 papers per year, with a consistently high level of research enthusiasm. The field demonstrates clinical efficacy for cancer-related pain, postoperative recovery, and labor pain. Research output remains limited to 843 publications. Thematic analysis post-2020 prioritized ICU settings, pediatric surgery, and dementia care. Critical gaps persist: standardized protocols, dose-response optimization, and mechanistic studies linking neurophysiological responses to outcomes (14–19). This change in research emphasis led to a significant increase in publications that year. Citation analysis indicates that three papers published in 2013 garnered a substantial number of citations from 2013 to 2018, signifying their considerable influence in the field. A randomized trial by Gutgsell et al. (20) demonstrated the effectiveness of music therapy interventions in alleviating pain in palliative care patients. A systematic review by Bradt et al. (21) investigated the effects of music therapy on patients with coronary artery disease, indicating potential benefits for anxiety, systolic blood pressure, heart rate, respiratory rate, sleep quality, and pain. However, the clinical significance of these results remains ambiguous. Özer et al. (22) examined physiological parameters, including blood pressure, heart rate, oxygen saturation, and respiratory rate, in 87 patients in a cardiovascular surgical intensive care unit, concluding that music therapy significantly alleviated pain-related physiological stress responses post-cardiac surgery. These seminal studies have established the groundwork for further research, facilitating the rise of prominent topics such as “colonoscopy,” “surgery,” relaxation,” “mood,” “music medicine,” and “palliative care.” Further extensively referenced literature, as depicted in Figure 11A, encompasses other foundational works that have influenced the field. The disproportionate citation impact of the 2013 cohort (4.2 citations/article/year) can be attributed to three converging factors. First, 11 of the 15 papers published that year focused on cancer-related or post-operative pain, two clinically high-priority areas that guaranteed broad readership. Second, nine of these papers appeared in journals with 2023 JCR impact factors ≥ 1.5 (e.g., Journal of Music Therapy, Pain Management Nursing), amplifying their visibility. Third, 73% of the 2013 articles involved bi-national authorship, predominantly US–Germany dyads, which increased the effective audience by 1.8-fold relative to single-country papers (χ^2^ = 4.92, *p* = 0.027). These conditions created a “perfect storm” of relevance, visibility and reach, explaining why 2013 became the field’s early citation inflection point.

In the last two decades, the field has seen an increasing number of contributing authors, with Bradt Joke, Silverman Michael J., Warth Marco, and Kessler Jens emerging as the most prominent researchers. These four scholars are the foremost in both H-index and publication output. Bradt Joke, associated with Drexel University in Pennsylvania, United States, has made substantial contributions to the utilization of music therapy for pain related to various conditions, including oncological pain, chronic pain, pain from brain injuries, and dental pain (23–26). His 2016 meta-analysis received significant attention in the music therapy and pain research community (27). This study determined that music therapy interventions significantly reduce anxiety, depressive symptoms, and pain in cancer patients. Recently, Bradt Joke has further investigated the role of music therapy in pain management for cancer patients, suggesting that its impact on chronic pain is influenced by autonomic nervous system function. He has also championed the enhancement of the discoverability and quality of music-based intervention information on publicly accessible cancer center websites (28). Silverman Michael J., associated with the University of Minnesota System, has concentrated on creating a therapeutic music program designed to enhance mood and alleviate pain in organ transplant recipients via a pilot study (29). Concurrently, Warth Marco of Ruprecht Karls University Heidelberg performed a systematic literature review on music therapy and cancer pain, providing a thorough synthesis of its use in palliative care (30). Kessler Jens and Warth Marco are members of the same research team at Ruprecht Karls University Heidelberg, a leading institution in this field. This institution, situated in Germany, engages in active collaboration with prominent research centers in the United States. These collaborations highlight the significance of institutional synergy in progressing the domains of music therapy and pain management. However, inter-institutional and international collaborations are still constrained, especially beyond the United States, China, and Germany. Turkey demonstrates a significant degree of domestic research collaboration, surpassing that of Germany. However, it falls short in international partnerships. Among 273 US articles, 62% involved only domestic institutions and only 9.3% included non-Anglophone partners. This pattern mirrors NIH funding regulations that privilege US-based PI leadership and indirect-cost returns to home institutions, inadvertently discouraging large-scale overseas coordination. Consequently, the top three US clusters (Harvard, Florida State, Ohio State) exhibit intercontinental link per 10 papers, whereas Germany’s central institutions (Heidelberg, Witten-Herdecke) average 2.4 such links (*p* < 0.01). Thus, fiscal and administrative structures—not scientific quality—appear to underlie the paradox of high US productivity yet constrained global embeddedness. Enhancing cross-national research collaborations could improve both the volume and caliber of research output in this domain. Institutional analysis indicates that Harvard University possesses the greatest quantity of publications among participating institutions. Drexel University serves as a pivotal research center, sustaining robust collaborative relationships with other institutions. The team, headed by Samuel N. Rodgers-Melnick at Case Western Reserve University, exhibits the most significant institutional affiliations. Future researchers examining music therapy and pain should pursue collaborative opportunities with these highly productive institutions. Furthermore, an analysis of journal sources reveals that the Journal of Music Therapy is the most cited (total citations = 912) and prolific publication (32 articles) in this discipline, achieving the highest rankings in both publication volume and H-index. Researchers seeking to publish their work may consult the list of premier journals depicted in Figure 6. Subscribing to these esteemed journals would grant access to the most recent advancements and innovative research in music therapy and pain management.

### 4.2 Research hotspots and trends in the field of music therapy and pain

Keywords represent fragmented data points, and a comprehensive analysis of these keywords can yield insights into the current research landscape and focal areas within the field. Through the analysis of keywords, we have discerned multiple nascent research focal points and trends within the domain, including “rehabilitation,” “dental anxiety,” “complementary medicine,” “integrative medicine,” “dementia,” “pediatric population,” “ill cancer patient,” “standardizing music characteristics,” “pregnant women,” “perioperative pain management,” and “virtual reality.” Through cluster analysis, we derived a set of keywords corresponding to four thematic categories: “music therapy,” “pain,” “mental health,” and “integrative medicine.” The following fields are summarized below.

### 4.3 Music therapy and pain topics

Music has been been shown to effectively reduce anxiety and improve mood in both medical and surgical patients, including those undergoing surgery and patients in intensive care units, across adult and pediatric demographics. Music is frequently used as a cost-effective intervention to mitigate surgical, acute, and chronic pain, while also improving the quality of life for patients in palliative care by fostering comfort and relaxation. Moreover, music therapy functions as an advantageous approach for cultivating empathy and compassion in carers. In the last 20 years, research in this domain has predominantly concentrated on subjects including “cancer patients,” “perioperative pain management,” “dementia,” “pediatric populations,” “dental anxiety,” and “pregnant women.” However, these studies are predominantly confined to systematic evaluations, reviews, and randomized controlled trials. Research on “cancer patients” is notably prominent among these topics. Li et al. (14) assessed 1,548 patients and determined that music therapy markedly enhanced overall quality of life scores by alleviating anxiety, depression, and pain in comparison to standard care, with the intervention lasting one to two months. Moreover, Bradt et al. (24) established that music therapy mitigated fatigue in cancer patients, whereas Tang et al. (31) noted its efficacy in enhancing sleep disorders post-chemotherapy in patients with small-cell lung cancer. In the realm of “perioperative pain management,” preoperative patients frequently encounter psychological anxiety, which, when addressed pharmacologically, can result in detrimental effects including respiratory complications, sedation, interference with anesthetic agents, and extended recovery durations. As a result, non-pharmacological methods such as music therapy, massage, and aromatherapy have become increasingly popular and are employed across various age demographics and medical conditions in surgical environments (32). A randomized controlled trial conducted by McClintock et al. (33) found that music therapy did not significantly alleviate perceived pain or anxiety during flexible cystoscopy, contradicting existing literature. This discrepancy may be due to variations in the ethnic composition of the study population or the lack of personalized music selection. Conversely, Huang et al. (34) examined the effects of music video therapy on early postoperative pain in preschool-aged children following cardiothoracic surgery. Their findings demonstrated that, in contrast to the control group, the music video therapy group displayed markedly reduced heart rate, mean arterial pressure, respiratory rate, frequency of postoperative patient-controlled analgesia (PCA) compressions, and sufentanil dosage. Additionally, pain scores evaluated with the Wong-Baker FACES (35) and FLACC (36) scales were markedly reduced in the music therapy group immediately following the initial intervention and at 1- and 2-days post-intervention, exhibiting a consistent decline in pain scores over time. Music therapy during the prenatal, labor, delivery, and postpartum phases has shown advantages for both mothers and infants in the treatment of pregnant women (37). These advantages encompass not only analgesia but also relaxation, diminished anxiety, alleviated psychosocial stress and depression, strengthened maternal-infant bonding, enhanced sleep quality, regulation of fetal heart rate and maternal blood pressure, and decreased postoperative medication consumption. Moreover, music therapy is extensively employed in pediatrics, hemodialysis, critical care medicine, dementia, and dentistry (38–42), where it has demonstrated beneficial effects on pain alleviation, anxiety mitigation, and physiological metrics. Although music therapy has been extensively utilized for numerous disorders in the last 20 years, research in this domain has primarily relied on clinical randomized controlled trials and meta-analyses. The effectiveness of these interventions is continually assessed through pain scales, anxiety scales, and vital sign measurements. Research has suggested that music therapy may lower cortisol levels in the body. However, the mechanisms underlying these effects remain insufficiently investigated. Future research must prioritize fundamental experimental studies to clarify these mechanisms and propel this field forward.

### 4.4 Research trends

In recent years, research on music therapy in pain management has shifted from descriptive analyses to mechanistic exploration and technological integration. The combination of virtual reality (VR) and music represents the forefront of this trend. When VR and music are combined, they may exert synergistic effects on pain: visual distraction from VR and auditory emotion regulation from music collectively target both sensory and emotional dimensions of pain (43) For example, palliative care patients undergoing VR-music interventions showed reduced pain intensity (Hedges’ g = −0.74) and improved physiological parameters (e.g., heart rate, oxygen saturation) (44). This effect is not solely due to distraction but also involves neuroplastic changes (e.g., modulation of auditory cortex-thalamus connections) (45, 46) and normalization of the autonomic stress response (47). Clinically, this integrated approach is particularly relevant for acute procedural pain (e.g., pediatric surgery, cancer treatment) and chronic pain conditions (e.g., fibromyalgia) (48, 49). Customization of VR music (e.g., patient-selected environments and music genres) optimizes individual responses (43), while technological accessibility (e.g., portable VR headsets) enhances scalability (45).

Complementary and alternative medicine modalities, including acupuncture, virtual reality, and aromatherapy, have surfaced as prominent focal points in this domain. Okutan et al. (50) examined the impact of virtual reality and musical interventions on patients post-laparoscopic abdominal surgery. Their findings demonstrated that the integration of music and virtual reality markedly alleviated pain and improved patient comfort, favorably affecting vital signs in contrast to the control group that did not undergo the intervention. Latchman et al. (51) conducted a comparison between acupuncture treatment alone and acupuncture in conjunction with music therapy for the management of cancer-related pain. Both groups demonstrated clinically and statistically significant reductions in pain intensity scores. However, disparities were noted in the duration of intervention effects and the allocation of benefits among age groups. Future research trends indicate that integrative medicine, along with complementary and alternative medicine, will remain central to this domain. Dental anxiety and rehabilitation have emerged as significant thematic trends in the past 2 years. Although prior studies have shown the effectiveness of music interventions in alleviating dental anxiety, the limitations of current research underscore the necessity for additional multicenter, large-sample, high-quality randomized controlled trials to produce more substantial clinical evidence (52). In this context, rehabilitation therapy primarily refers to the application of music interventions during pregnancy or the perioperative phase (53, 54). However, when music therapy is combined with acupuncture, no super-additive analgesic effect was observed (51), highlighting the need to delineate indications within multimodal protocols. Furthermore, there are three persistent limitations in the current evidence base: (1) absence of dose–response standardization, (2) scarcity of cross-cultural replication beyond North America and Europe, and (3) over-reliance on subjective outcome scales. Future standardization efforts must therefore be built on four pillars—collaborative guideline drafting, evidence-based reporting, context-adaptable flexibility, and continuous quality evaluation—so that music therapy can evolve from an adjunctive modality into a globally accepted, evidence-based pillar of integrative healthcare. The selection of music for therapeutic purposes is predominantly subjective, reliant on individual preferences, and there is currently no standardized treatment protocol concerning the duration of therapeutic interventions. A restricted quantity of studies has indicated decreases in chronic pain and fibromyalgia following standardized exposure to Mozart’s music. Nonetheless, additional longitudinal studies with larger sample sizes are required to corroborate these findings and draw more conclusive results (18).

### 4.5 Critical reflection and real-world translational gaps

While bibliometric data indicate a 2.3-fold increase in publication output since 2020, a pronounced disconnect persists between evidence quality and insurance coverage. The majority of “music therapy” trials continue to employ one-size-fits-all playlists that disregard patients’ idiosyncratic musical preferences and cultural backgrounds, thereby attenuating effect sizes. Unless large-scale, low-risk-of-bias randomized controlled trials formally map dose–response relationships between patient-selected music and clinically relevant endpoints, the intervention will remain classified as experimental and will not overcome systemic reimbursement barriers.(55)

Second, the intervention repertoire is overwhelmingly Western-centric: 71% of studies use Western music, whereas only 6% incorporate non-Western pieces preferred by participants (56), an imbalance that dilutes efficacy estimates in culturally diverse samples and probably contributes to the high heterogeneity. Future protocols should therefore be prospectively registered with culturally adaptive designs aligned with the World Health Organization 2021 arts-and-health framework (56).

Third, although terms such as “neuroplasticity” and “Heart Rate Variability” appear frequently in the literature, systematic validation of dose-dependent functional connectivity within relevant neural circuits (e.g., basal ganglia-motor cortex) is lacking. The enhanced clinical benefit associated with personalized music may hinge on recruitment of the autobiographical-memory-limbic-reward network. Accordingly, subsequent RCTs should incorporate neuroimaging secondary endpoints to clarify the causal chain linking preference, neural response, and symptom relief (57).

## 5 Limitations

This study conducted visual analysis using bibliometric software, mainly based on the WOSCC database, supplemented by PubMed for clinical trials. While WOSCC is a prevalent and robust database for bibliometric analysis and PubMed adds clinical focus, we did not perform cross-database deduplication. Thus, any overlapping records between WOSCC and PubMed may have been counted twice, potentially inflating certain bibliometric indicators and introducing bias into our results. For differences in citation formats across databases and inconsistent information extraction by the software made it impractical to integrate data from Scopus or other sources into the present study, further restricting the comprehensiveness of the evidence, base and introducing potential biases, such as publication bias. This could skew our analysis toward over-represented findings and potentially overlook studies with negative or inconclusive results. Secondly, the search strategy was restricted to English-language publications and to the specific terms listed in the method. Consequently, relevant studies published in other languages or indexed with alternative keywords may have been missed, potentially limiting the comprehensiveness of our analysis. Thirdly, due to the continuous updates of the databases, the findings of this bibliometric analysis inherently represent a snapshot in time and may not reflect the very latest publications or emerging trends at the time of writing. Fourth, although the study was designed in accordance with general research standards, the absence of a formal risk-of-bias assessment limits the reliability of our findings. Furthermore, the analytical software used (CiteSpace, VOSviewer, Bibliometrix) has inherent constraints: it cannot automatically disambiguate author names across different formats, aggregate contributions from all co-authors of an article with equal weight in network analyses, or assess the methodological quality or risk of bias within the included studies. This study focused exclusively on delineating research trends and focal points in music therapy and pain over the past two decades. A potential limitation of bibliometric methods is that recently published, potentially significant articles with lower current citation counts may be under-represented in analyses like co-citation networks or burst detection. Finally, we acknowledge that bibliometric indicators—particularly citation counts and h-indices—may overestimate the scientific or clinical impact of individual studies. High citation frequency does not equate to methodological rigor or direct patient benefit. Therefore, citation-based rankings should be interpreted as proxies for academic visibility rather than definitive measures of therapeutic value. Future research should consider employing more comprehensive search strategies, including the use of additional databases and the incorporation of alternative search terms, to ensure a more comprehensive and unbiased analysis. Despite these limitations, our study provides a comprehensive overview of the research trends and focal points in music therapy and pain management over the past two decades. We hope that our findings will serve as a valuable foundation for future research and clinical applications in this field.

## 6 Conclusion

This study represents the first comprehensive bibliometric analysis spanning two decades (2004–2024) on the application of music therapy in pain management, utilizing WOSCC and PubMed data visualized through CiteSpace, VOSviewer, and R software. Key findings reveal: (1) a substantial and accelerating annual growth in publications, particularly post-2020; (2) the United States, China, and Germany as dominant contributors, though international collaboration remains concentrated and requires expansion; (3) core authors (e.g., Bradt Joke, Silverman Michael J.) and leading journals (e.g., Journal of Music Therapy) shaping the field; and (4) a clear thematic evolution from foundational topics like anxiety and cancer pain toward emerging frontiers such as rehabilitation, virtual reality, dementia care, pediatric applications, and integrative medicine approaches. The analysis underscores persistent gaps, notably the lack of standardized protocols for music selection and intervention delivery, insufficient mechanistic understanding linking neurophysiological responses to clinical outcomes, and the need for optimized dose-response relationships. The findings propose a roadmap for future endeavors: optimizing the clinical translation of music therapy requires addressing these standardization and mechanistic gaps; enhancing the quality and volume of research output necessitates fostering broader cross-national and inter-institutional collaborations; and exploring synergistic effects with other complementary therapies warrants further investigation. This study provides significant insights and guidance for researchers and clinicians, highlighting both the established value and the future directions essential for advancing the field of music therapy in pain management.

## Data Availability

The original contributions presented in this study are included in this article/Supplementary material, further inquiries can be directed to the corresponding author.

## Supplementary material

Supplementary Table 1 Top 10 journals contributing to music-therapy and pain research (2004-2024) ranked by number of publications, 2023 JCR Impact Factor (IF) and quartile.
