# Supplementary material for: A dual-database bibliometric analysis of music-based interventions and pain from 2004 to 2024
Source: Front Med (Lausanne). 2025 Oct 16;12:1671234. doi: 10.3389/fmed.2025.1671234 (PMC12571727; doi:10.3389/fmed.2025.1671234)
Supplement: Supplementary Table 1 — Top 10 journals contributing to music-therapy and pain research (2004-2024) ranked by number of publications, 2023 JCR Impact Factor (IF) and quartile. [file Table_1.docx]

**Supplementary Table 1 Top 10 journals contributing to music-therapy and pain research (2004-2024) ranked by number of publications, 2023 JCR Impact Factor (IF) and quartile.**

| **Rank** | **Journal** | **Publications** | **Country** | **IF 2023** | **JCR Quartile** |
| --- | --- | --- | --- | --- | --- |
| 1 | Journal of Music Therapy | 32 | USA | 1.9 | Q3 |
| 2 | Arts in Psychotherapy | 19 | England | 1.5 | Q2 |
| 3 | Complementary Therapies in Medicine | 17 | England | 3.3 | Q1 |
| 4 | Supportive Care in Cancer | 17 | Germany | 3.1 | Q1 |
| 5 | Pain Management Nursing | 15 | USA | 1.7 | Q2 |
| 6 | Journal of Pain and Symptom Management | 14 | USA | 4.7 | Q1 |
| 7 | BMC Complementary Medicine and Therapies | 12 | England | 3.9 | Q2 |
| 8 | Nordic Journal of Music Therapy | 11 | Norway | 1.6 | Q3 |
| 9 | Music Therapy Perspectives | 10 | USA | 1.2 | Q3 |
| 10 | Journal of Pediatric Oncology Nursing | 9 | USA | 1.7 | Q2 |
